# Post‐Synthetic Cerium Modification of Commercial NCM811 for Concurrent Cycling and Safety Upgrading

**DOI:** 10.1002/advs.78626

**Published:** 2026-11-03

**Authors:** Wei‐Wei Wang, Da‐Kun Chen, Wei Kang, Xiao‐Mei Wu, Qing Cao, Quan‐Fang Wang, Si‐Yuan Ma, Hai‐Long Wang, Jing‐Hua Tian, Zhong‐Qun Tian

**Affiliations:** ^1^ Innovation Laboratory for Sciences and Technologies of Energy Materials of Fujian Province (IKKEM) Xiamen China; ^2^ State Key Laboratory of Physical Chemistry of Solid Surfaces iChEM College of Chemistry and Chemical Engineering Xiamen University Xiamen China

**Keywords:** cerium, commercial NCM811, lithium‐ion battery, oxygen release suppression, synergistic modification

## Abstract

The widespread use of Ni‐rich LiNi_0.8_Co_0.1_Mn_0.1_O_2_ (NCM811) cathodes in high‐energy batteries remains limited by structural degradation, side reactions, and oxygen release, yet most modification strategies prove incompatible with commercial materials. This work presents a facile solid‐state method that achieves synergistic cerium ion (Ce^3+^) doping and CeO_2_ coating on commercial NCM811 by mixing with CeO_2_ nanoparticles followed by calcination. Structural and morphological analyses confirm Ce incorporation into the near‐surface lattice and the formation of a nanoscale CeO_2_ layer without disturbing the layered framework. Electrochemical tests demonstrate that the optimal 1 wt.% Ce‐modified sample exhibits superior rate capability and long‐term stability, retaining 82.04% of its initial capacity after 500 cycles at 1 C, compared to 74.10% for pristine, owing to suppressed cation mixing, enhanced structural stability, and improved Li^+^ diffusion kinetics. Crucially, differential electrochemical mass spectrometry shows Ce modification curtails O_2_ release and reduces H_2_ and CO_2_ generation across various rates, effectively mitigating gas evolution associated with safety degradation. Accelerating rate calorimetry further confirms delayed thermal runaway and improved adiabatic stability. Overall, this work establishes a scalable post‐treatment route to simultaneously boost the electrochemical performance and safety of commercial nickel‐rich cathodes, offering a practical solution for next‐generation lithium‐ion batteries.

## Graphical abstract

Cerium ion doping and CeO_2_ coating work synergistically to reinforce commercial NCM811 cathodes. This strategy stabilizes the lattice and strengthens the interface against degradation and side reactions. The modified cathode shows enhanced rate capability and cycling stability. Notably, suppressed gas release improves safety, making this scalable method a promising route for advanced batteries.

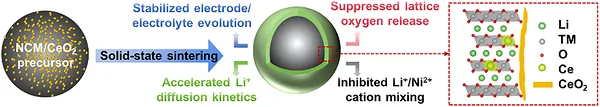

## 1 Introduction

As modern technology evolves, batteries have undergone constant development to meet the power needs of diverse applications, from large transport vehicles to portable electronic devices [1]. Lithium‐ion batteries (LIBs) have become indispensable for sustainable electrochemical energy storage, supporting applications ranging from electric vehicles and portable electronics to grid‐scale systems, and playing a crucial role in the global transition to sustainable energy [2, 3, 4, 5]. Owing to their relatively high specific capacity, high‐nickel lithium layered oxide cathodes (LiNi_x_Co_y_Mn_1−x−y_O_2_, x ≥ 0.8) are viewed as the most promising candidates [6, 7, 8, 9]. In particular, the LiNi_0.8_Co_0.1_Mn_0.1_O_2_ (NCM811) cathode has emerged as a leading candidate for high‐energy‐density batteries due to its high specific capacity and cost‐effectiveness [10, 11, 12, 13]. However, its widespread application is hindered by inherent challenges including structural degradation, severe interfacial side reactions, and lattice oxygen release during cycling, leading to rapid capacity fade and serious safety risks [14, 15, 16].

Various modification strategies, including elemental doping, surface coating, and morphology optimization, have been explored to mitigate these limitations [17, 18, 19, 20, 21]. Luo et al. synthesized zirconium (Zr)‐gradient NCM811 cathodes via a simulation‐guided flame‐assisted spray pyrolysis method [22]. Benefiting from the solubility‐driven Zr‐rich shell, the optimized cathode exhibited a capacity retention of 83.04% after 100 cycles at 0.5 C, effectively suppressing the H2→H3 phase transition and enhancing structural stability. Similarly, Ying et al. prepared bulk boron (B) doped NCM811 using a co‐precipitation method and verified that B occupies tetrahedral sites in the Li layer. The inductive effect of B─O bonds weakens transition metal (TM)─O covalency, delays structural collapse, and reduces oxygen release, resulting in a high initial capacity of 210 mAh g^−1^ and a capacity retention of 95.3% after 100 cycles [23]. Moreover, according to the results of Wang et al., the polyacrylonitrile/Zeolitic Imidazolate Framework‐8 (cPAN/ZIF‐8) dual‐coating effectively stabilized the cathode‐electrolyte interphase (CEI) of Ni‐rich NCM811 [24]. Compared with pristine NCM811, the modified sample exhibited much lower polarization, smaller charge‐transfer resistance, and superior structural integrity upon cycling. These works have effectively improved the electrochemical properties of NCM cathode to some extent.

Rare earth elements, owing to their high charge and large ionic radii, exhibit significant advantages in the modification of cathode materials. Among them, cerium (Ce) is widely recognized as an effective modifier for cathode materials [25, 26], and a large number of relevant studies have demonstrated that it can improve the structural stability and cycle life of cathode materials [27, 28]. Ce ions can strongly bind to lattice oxygen, stabilizing the bulk structure and suppressing oxygen loss, while a CeO_2_ coating can serve as a protective barrier against electrolyte side reactions [29]. Using density functional theory calculations, Deng et al. found that Ce modification decreased the bandgap of LiNi_0.92_Co_0.04_Mn_0.04_O_2_ from 2.0627 to 0.9156 eV, greatly improving its electronic conductivity, while simultaneously reducing the Li^+^ diffusion energy barrier from 0.66 to 0.37 eV [30]. Thus, a combined Ce‐doping and CeO_2_ coating strategy offers a promising route to synergistically enhance both the bulk and surface properties of NCM811. Wu et al. further reported that Ce doping not only mitigated Li^+^/Ni^2+^ mixing by oxidizing Ni^2+^ to Ni^3+^, but also reinforces the Ce─O bonding. Furthermore, a nanoscale CeO_2_ coating protected the surface from electrolyte corrosion, thereby improving the electrochemical performance and high‐voltage stability of NCM811 [31]. This internal‐external synergistic effect greatly enhances structural stability and rate capability at high voltages.

Doubts about the safety of Ni‐rich cathodes persist in both industry and academia, restricting their further application [32, 33, 34, 35]. However, it is worth noting that most previous studies have primarily focused on the electrochemical performance indicators [24, 36, 37, 38], lacking exploration of the interconnections among interrelated issues such as gas release and thermal stability of the modified cathode materials during cycling, which are crucially important for battery safety [39, 40, 41, 42, 43]. Another critical issue is that, to the best of our knowledge, most existing methods are applied during material synthesis [44, 45, 46], making them unsuitable for the further modification of mass‐produced commercial NCM811. Despite dominating the current market, these commercial materials often fail to meet the stringent demands for long‐term cycling stability and safety in advanced applications. Therefore, developing effective, scalable post‐treatment methods for upgrading commercial NCM811 represents an important yet underexplored research direction.

In this study, a simple and efficient post‑synthetic solid‑state treatment strategy is developed to achieve synchronous Ce‐doping into the near‑surface lattice and CeO_2_ coating on commercial NCM811 cathode materials (Figure 1). The successful modification is systematically confirmed by a combination of characterization techniques, including X‐ray diffraction with Rietveld refinement, X‐ray photoelectron spectroscopy, scanning and transmission electron microscopy, and energy‐dispersive X‐ray spectroscopy mapping, which collectively verify the retention of the layered structure, the gradient distribution of Ce from surface to bulk, and the formation of a nanoscale CeO_2_ protective layer. Electrochemical evaluations demonstrate that the optimally modified cathode (1 wt.% Ce) delivers significantly improved rate capability and cycling stability, maintaining a high‐capacity retention of 82.04% after 500 cycles at 1 C, in contrast to only 74.10% for the pristine NCM811. Moreover, differential electrochemical mass spectrometry reveals that the modified material drastically suppresses O_2_ release and markedly reduces the evolution of H_2_ and CO_2_ across various current rates, while accelerating rate calorimetry further confirms delayed thermal runaway and enhanced adiabatic safety. This work not only provides a scalable post‑treatment route to upgrade commercially available NCM811, but also highlights the synergistic internal‑external effects of Ce modification, offering a promising and practical strategy for developing high‑performance and intrinsically safer nickel‑rich cathodes for advanced lithium‑ion batteries.

**FIGURE 1:**
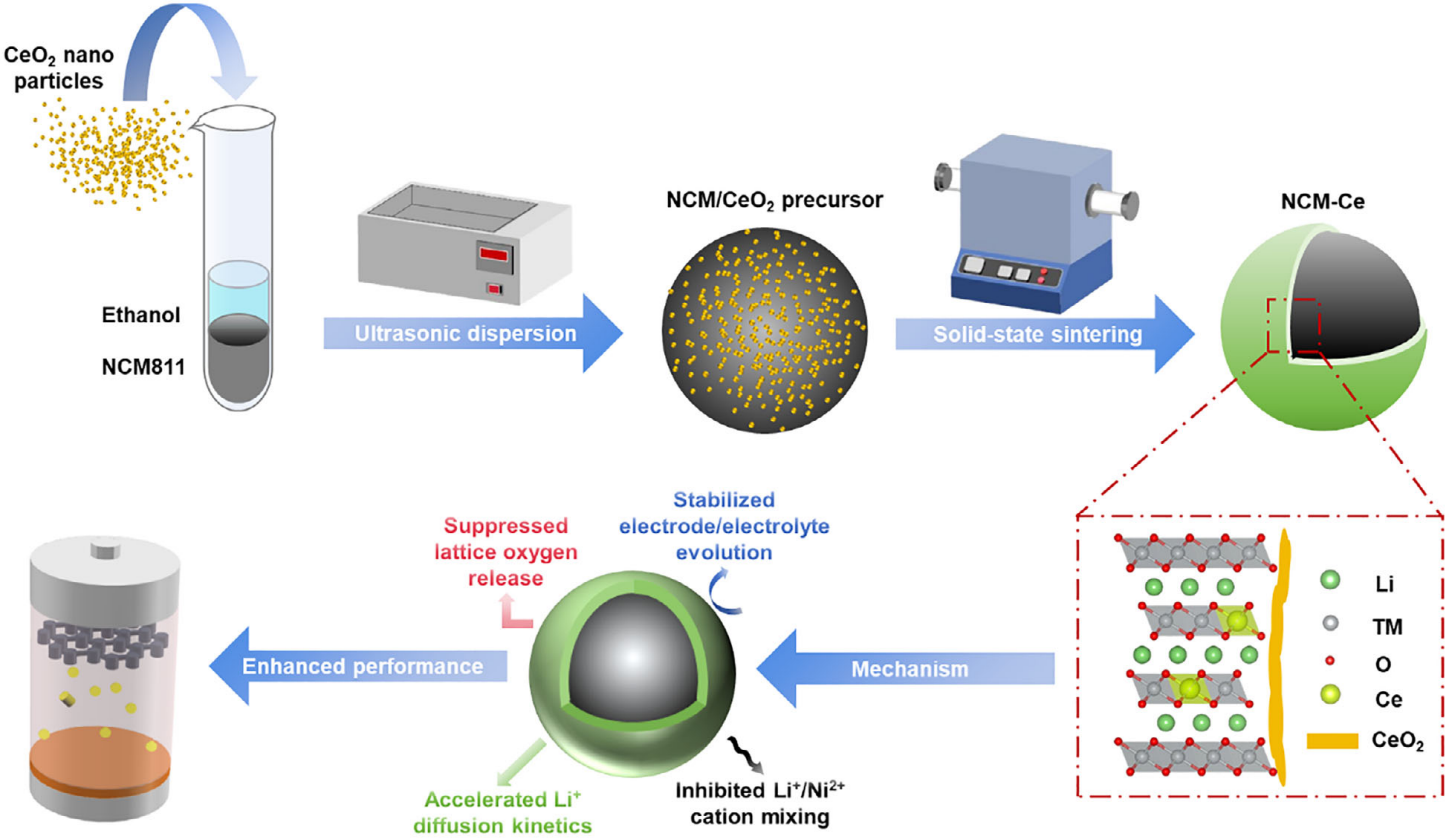
Schematic illustration of the synthesis process for the Ce modified NCM cathode materials.

## 2 Results and Discussion

### 2.1 Structural and Morphology Characterization

A set of Ce‐modified NCM materials were synthesized for preliminary optimization. As displayed in Figure S1, no diffraction peaks assigned to CeO_2_ can be detected in the X‐ray diffraction (XRD) patterns at low doping concentrations, such as 0.5%. A new diffraction peak emerges at about 28.8° when the CeO_2_ content reaches 1%, which corresponds to the (111) crystal plane of CeO_2_. Meanwhile, the SEM images in Figure S2 clearly reveal distinct CeO_2_ nanoparticles remaining on the surface of modified NCM materials as the Ce content further increased. Further electrochemical performance tests were conducted on the cathode materials with different Ce doping amounts, as shown in Figure 2. The discharge capacities at both 0.1 C and 1 C initially increased and subsequently decreased with increasing Ce content. The material with 1% Ce modification exhibits the largest discharge capacity, reaching 208.70 mAh g^−1^ at 0.1 C and 189.70 mAh g^−1^ at 1 C. EIS measurements on activated cells with different Ce contents show similar R_s_ and R_SEI_ but markedly different R_ct_ (Figures S3 and S4). The 1% Ce sample exhibits the lowest R_ct_ (59.88 Ω), whereas the 1.5% Ce and 2% Ce samples show much higher values (107.92 and 109.13 Ω). This indicates that the capacity decline observed at modification levels of 1.5% and 2% may be related to excessive surface CeO_2_, which forms a thicker insulating layer that impedes Li^+^ transport and lowers the lithium storage performance of the cathode material.

**FIGURE 2:**
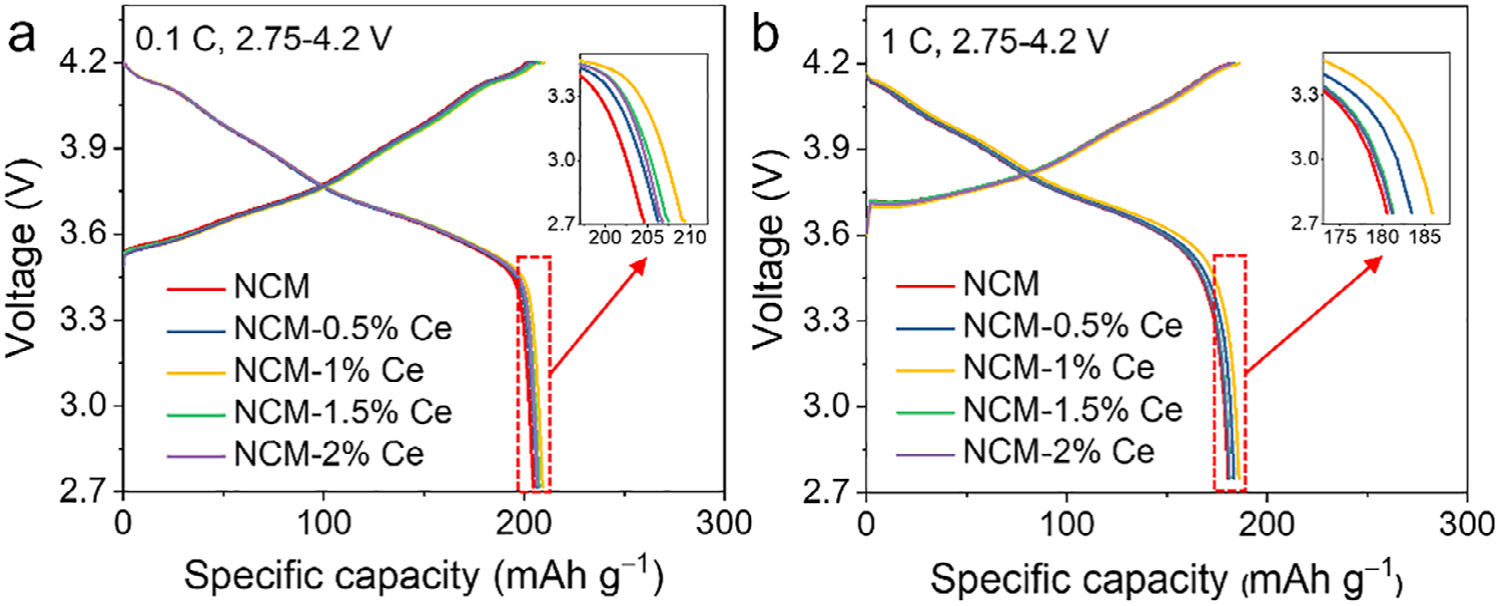
Charge and discharge profiles of the NCM samples with different Ce contents at (a) 0.1 C and (b) 1 C in NCM||Li half coin cells.

At 0.5% Ce, the CeO_2_ domains are amorphous or ultra‐small and thus below the XRD detection limit, while Ce^3+^ incorporates into the near‐surface lattice. At 1%, this lattice doping approaches saturation, and excess Ce precipitates as crystalline CeO_2_, producing the (111) peak. This apparent threshold coincides with the optimal synergy. Specifically, a moderate CeO_2_ coating protects the interface, whereas higher Ce contents produce excessive surface CeO_2_, which increases interfacial resistance and degrades performance. To investigate the effect of high‐temperature calcination on the lithium content, inductively coupled plasma‐optical emission spectrometry (ICP‐OES) measurements were performed on both pristine NCM and NCM‐1% Ce. As shown in Table S1, only a slight decrease in Li content was observed, from 7.15 to 7.04 wt.%, and the Li/TM ratio remained nearly unchanged, confirming that Li volatilization was negligible. The actual Ce content was 0.79 wt.%, in good agreement with the nominal value of 1 wt.%. Through a comprehensive comparison, the optimal Ce modification concentration was determined to be 1 wt.%.

As shown in Figure 3a, the diffraction patterns of both pristine and Ce‐modified NCM samples are in good agreement with the standard PDF card (PDF#09‐0063) of the layered α‐NaFeO_2_‐type structure, indicating that the Ce‐based modification does not alter the original layered crystal framework of the pristine NCM. Meanwhile, as displayed in Figure S5a, the (003) diffraction peak exhibits a slight shift to higher 2θ angles after Ce modification, which suggests a slight shrinkage of the crystal lattice. Furthermore, the Rietveld‐refined XRD patterns presented in Figure 3b,c and Figure S6 and Tables S2–S4 provide quantitative evidence for the variation in cation ordering in the material's crystal structure. For Ni‐rich cathode materials, the I(003)/I(104) intensity ratio is generally associated with Li^+^/Ni^2+^ antisite defects and the preferential growth of primary particles [47, 48, 49, 50, 51]. However, we found that the I(003)/I(104) ratio decreases from 1.08 to 0.96 after Ce modification, primarily due to the differential attenuation of the (003) reflection by the surface CeO_2_ coating rather than an increase in bulk cation mixing. Rietveld refinement confirms that the bulk Li^+^/Ni^2+^ mixing actually decreases from 3.13% to 1.07%, demonstrating that the layered framework is better ordered after Ce modification. X‐ray absorption fine structure (XAFS) spectroscopy analysis (Figure S7) further supports this lattice shrinkage. After Ce modification, the Ni *K*‐edge shifts by ∼0.6 eV to higher energy, confirming Ni^2+^ oxidation to Ni^3+^ as charge compensation for Ce^4+^ reduction to Ce^3+^, while Co and Mn edges remain unchanged. EXAFS reveals a shorter Ni─O distance, indicating Ni─O bond contraction. Since Ni^3+^ is smaller than Ni^2+^, this in‐plane TM─O contraction dominates over expansion from larger Ce^3+^, explaining the slightly reduced lattice parameters observed by XRD.

**FIGURE 3:**
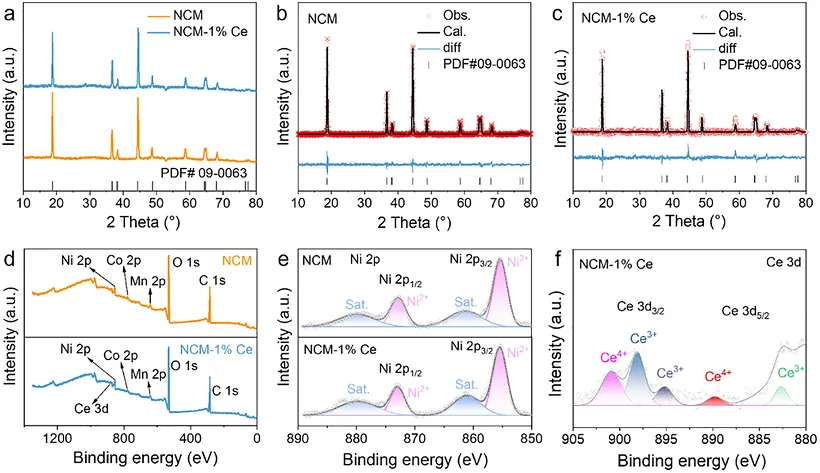
(a) XRD patterns of the pristine NCM and NCM‐1% Ce. Rietveld refinements of (b) the pristine NCM and (c) NCM‐1% Ce. Comparisons of the (d) XPS survey spectra and (e) Ni 2p high‐resolution XPS spectra of the pristine NCM and NCM‐1% Ce. (f) Ce 3d XPS spectrum of NCM‐1% Ce.

XPS analysis was conducted to investigate the surface chemical states of the materials. As presented in Figure 3d, the Ni 2p, Co 2p, Mn 2p, O 1s and C 1s spectra of the NCM‐1% Ce sample show no discernible shift relative to the pristine NCM. Further examination (Figure 3e and Figure S5b,c) reveals that, the Ni 2p and Co 2p spectra remain essentially unchanged, whereas the Mn 2p spectrum exhibits a slight increase in binding energy, suggesting a minor alteration in the chemical environment of Mn species after Ce modification. This moderate electronic modulation helps stabilize the surface structure and suppress transition metal dissolution, which is beneficial for the electrochemical stability of the cathode material [30]. Notably, the survey spectrum of the modified sample displays an additional Ce 3d signal (Figure 3d). The high‐resolution Ce 3d spectrum (Figure 3f) can be deconvoluted into characteristic spin‐orbit doublets corresponding to both Ce^3+^ and Ce^4+^ species, indicating the coexistence of Ce^3+^ and Ce^4+^ on the surface of the NCM‐1% Ce sample. Combined with the previous study results, the Ce^4+^ mainly exists in the form of a CeO_2_ coating layer on the modified material surface, while Ce^3+^ is likely doped into the bulk NCM lattice. XPS depth profiling (Figure S8) further clarifies the distribution of Ce species. At the surface (0 nm), a weak Ce^3+^ signal accompanies the dominant Ce^4+^ signal, consistent with a CeO_2_ coating that is not fully dense or uniform [52]. After sputtering to 10 nm, the Ce^3+^ signal increases markedly while Ce^4+^ remains clearly present, indicating that Ce^3+^ becomes enriched in the near‐surface region as the coating is gradually removed. At 20 nm, Ce^3+^ persists with peak broadening, whereas Ce^4+^ gradually decreases, likely due to uneven coating thickness. These results indicate that Ce species extend beyond the outermost surface into the near‐surface region (> 10 nm), consistent with Ce^3+^ incorporation into the near‐surface lattice. Ce^3+^ originates from interfacial solid‐state redox during calcination, where Ce^4+^ oxidizes near‐surface Ni^2+^ to Ni^3+^ and is itself reduced to Ce^3+^. This Ce^3+^ incorporates into the lattice, while the reduced Ni^2+^ concentration suppresses Li^+^/Ni^2+^ mixing [34]. XPS results demonstrate that Ce‐modification does not significantly alter the surface transition metal environment of NCM811 material and successfully introduces mixed‐valence Ce species, realizing a combined structure featuring Ce^3+^ doping into the near‐surface bulk lattice and a CeO_2_ coating on the particle surface.

The morphology and microstructure of the pristine NCM and NCM‐1% Ce samples were investigated by SEM and TEM, as shown in Figure 4. The SEM images in Figure 4a,b reveal that both samples consist of spherical secondary particles, with diameters ranging from 5 to 20 µm, which are agglomerates of nano‐sized primary particles. No significant morphological change was observed after modification, confirming that the post‐synthetic treatment preserves the overall particle structure. High‐resolution TEM (HRTEM) analysis provides direct evidence for the formation of the Ce coating layer. As displayed in Figure 4c–e and Figure S9a, lattice fringes corresponding to the (104) plane (*d* = 0.204 nm) of the layered NCM structure are clearly resolved in pristine NCM sample. Critically, in the modified sample (Figure 4f–h and Figure S9b), an additional set of fringes with a d‐spacing of 0.311 nm was detected on the particle surface, corresponding to the (111) plane of CeO_2_. This confirms the in situ formation of a conformal CeO_2_ coating layer (∼10 nm thick) comprising nano‐crystalline domains. Furthermore, as shown in Figure 4i, the corresponding EDS mappings demonstrate a homogeneous distribution of Ni, Co, Mn, Ce, and O elements in the modified NCM particles.

**FIGURE 4:**
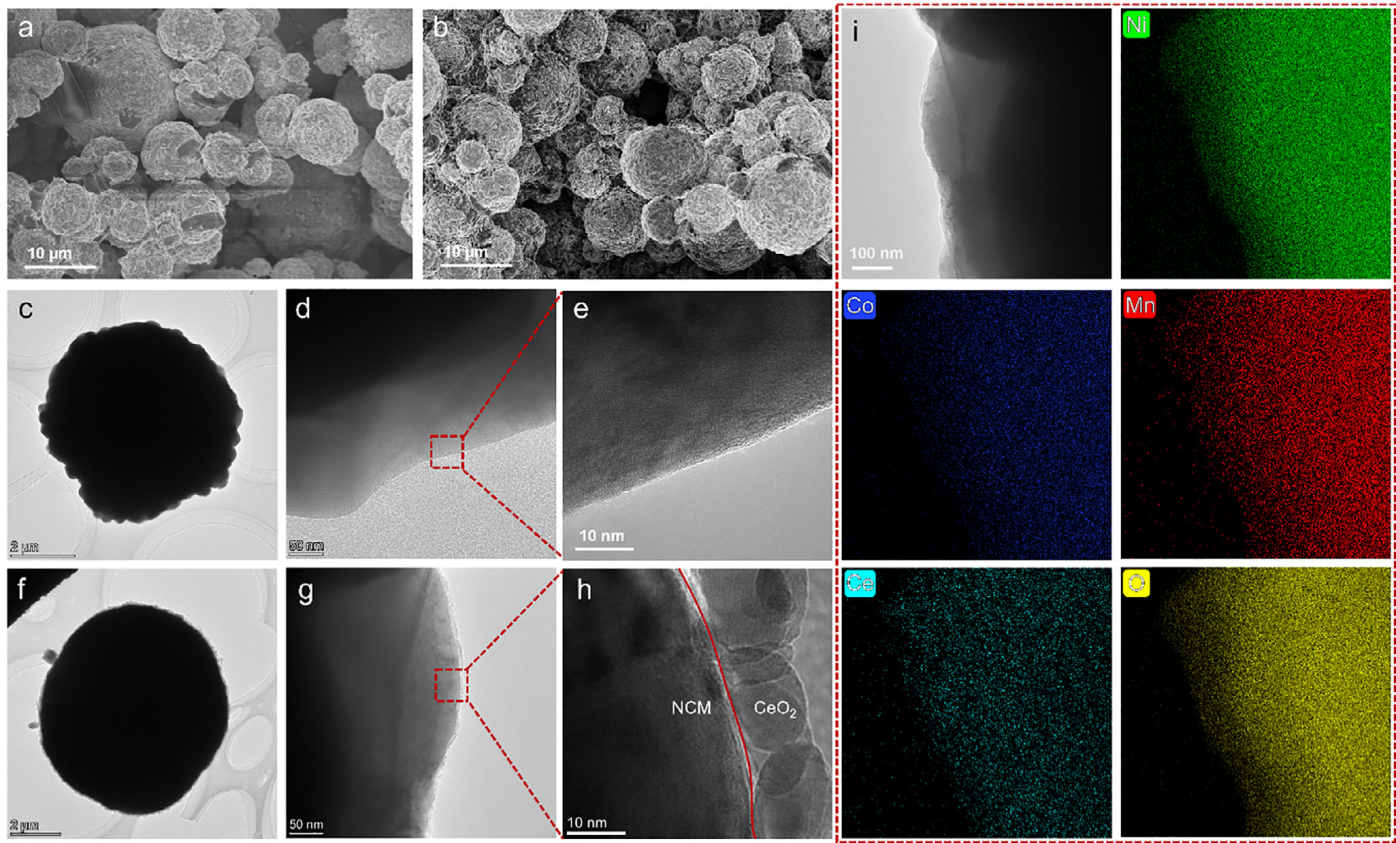
SEM images of the (a) pristine NCM and (b) NCM‐1% Ce. HRTEM images at different magnifications of the (c–e) pristine NCM and (f–h) NCM‐1% Ce. (i) EDS elemental mapping of NCM‐1% Ce.

### 2.2 Electrochemical Performance

The discharge capacities of the pristine NCM and NCM‐1% Ce samples were evaluated within a voltage range of 2.75–4.2 V. As shown in Figure 5a, at a current density of 0.1 C, the discharge capacity of the NCM‐1% Ce reaches 201.05 mAh g^−1^, which is higher than the 192.99 mAh g^−1^ delivered by the pristine NCM. Meanwhile, Figure 5b indicates the discharge capacity at 1 C is also improved from 176.77 mAh g^−1^ for the pristine NCM to 188.38 mAh g^−1^ for the NCM‐1% Ce sample, indicating the enhanced electrochemical activity and Li^+^ utilization efficiency after Ce modification. The long‐term cycling stability was evaluated at 1 C, and the results are presented in Figure 5c. The NCM‐1% Ce cathode demonstrates significantly enhanced capacity retention. After 500 cycles, it maintains a discharge capacity of 154.55 mAh g^−1^, corresponding to a high retention of 82.04%. In contrast, the pristine NCM cathode suffers from severe capacity fade, retaining only 130.99 mAh g^−1^ (74.10% retention). Moreover, Figure 5d indicates that the discharge curve of the NCM‐1% Ce cathode after 500 cycles shows a reduced voltage polarization and a higher median voltage than the pristine NCM. The improved discharge capacity and long‐term cycling performance of the NCM‐1% Ce cathode material can be potentially attributed, on the one hand, to the better structural stabilization and reduced cation mixing induced by bulk Ce doping, and on the other hand, to the effective suppression of parasitic reactions between the cathode and electrolyte by the surface coating layer of CeO_2_. The reduced Li^+^/Ni^2+^ disorder induced by Ce incorporation is expected to improve structural reversibility, while the CeO_2_ coating can mitigate direct contact between the cathode surface and electrolyte, thereby alleviating interfacial degradation during cycling. The superior rate capability of the NCM‐1% Ce cathode is evident from the tests at various current densities, as presented in Figure 5e. It consistently delivers higher capacities than the pristine NCM cathode at all rates, with the difference becoming more pronounced at high rates. For instance, the modified cathode delivers 155.39 mAh g^−1^ at 5 C, retaining 79.26% of its capacity at 0.2 C. Under the same condition, the pristine NCM cathode only achieves 144.63 mAh g^−1^, with a lower retention of 75.21%.

**FIGURE 5:**
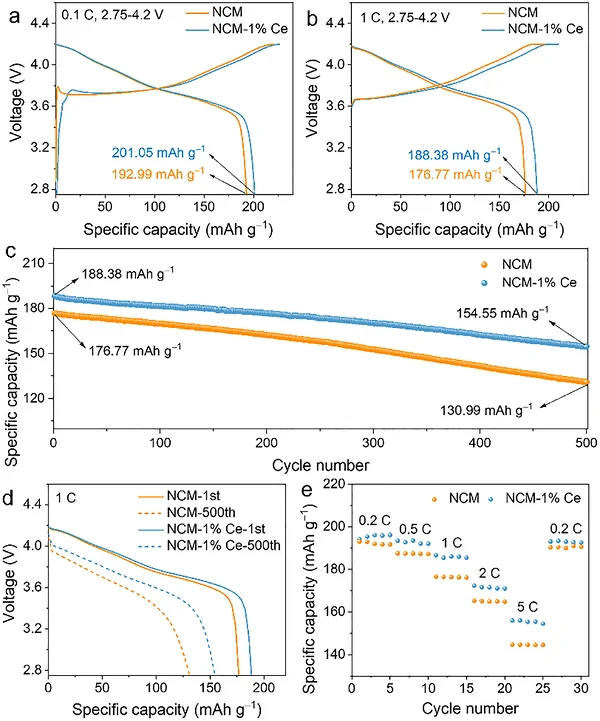
Initial charge and discharge profiles at (a) 0.1 C and (b) 1 C. (c) Long‐term cycling performance of the pristine NCM and NCM‐1% Ce materials at 1 C. (d) Discharge profiles at the first and 500th cycles and (e) rate capability of the pristine NCM and NCM‐1% Ce.

Cycling tests at a higher cutoff voltage of 4.5 V and elevated temperature of 60°C were performed to demonstrate the benefits of Ce modification (Figure S10). At 4.5 V, NCM‐1% Ce delivered 215.50 mAh g^−1^, about 5 mAh g^−1^ higher than pristine NCM. After 100 cycles, the modified cell retained 89.37% of its initial capacity, whereas the pristine cell faded rapidly after 20 cycles and retained only 67.71%. At 60°C, discharge capacities changed little, and the modified material showed a capacity retention of 93.37%, higher than that of pristine NCM (90.70%). To verify the general applicability of the post‐synthetic modification strategy, we applied the same protocol, namely 1 wt.% Ce doping and calcination at 550°C for 12 h, to another commercial NCM‐R material. As shown in Figure S11, the initial discharge capacity at 0.1 C increases from 204.17 to 209.04 mAh g^−1^. Meanwhile, both the rate capability and long‐term cycling performance are significantly enhanced. After 500 cycles, the capacity retention of the modified material improves from 81.41% to 85.29% compared with that of the unmodified material. These consistent improvements confirm that our Ce modification strategy is not limited to a specific product but is universally applicable to a variety of commercial NCM cathodes.

This enhanced rate capability can be attributed to the improved Li^+^ diffusion kinetics and the reduced electrochemical polarization induced by Ce modification. As shown in Table S5, the ionic radii of different metal ions are compared. Although Ce^3+^ possesses a larger ionic radius than the transition metal ions in the NCM lattice, the lattice parameters exhibit a minor decreasing trend upon modification. The proposed mechanism is schematically illustrated in Figure S12: the strong Ce─O bonds and shorter radial distance of Ni─O bonds (Figure S7) formed upon Ce incorporation induce an in‐plane contraction of the transition metal oxide (TMO_2_) layers, reducing the interlayer spacing from 2.206 to 2.106 Å, while also locally expanding the two‐dimensional Li^+^ diffusion channels between the oxygen layers, with the channel size increasing from 2.538 to 2.611 Å. Although the Rietveld refinement results reveal a slight decrease in the lattice parameter *c*, this macroscopic average structural change does not impede the rapid Li^+^ transport through the local channels, thereby further boosting the rate capability of the NCM‐1% Ce material.

To probe the structural evolution during cycling, post‐mortem SEM analysis was conducted on the electrodes after long‐term cycling. As shown in Figure S13, the cycled pristine NCM electrode suffers from severe structural degradation, where the spherical secondary particles are extensively fractured with severe internal microcracks. In contrast, the NCM‐1% Ce electrode largely retains its original spherical morphology and particle integrity, with significantly mitigated crack formation. This remarkable difference in structural resilience can be attributed to the synergistic effect of bulk Ce^3+^ doping and the surface CeO_2_ coating. The Ce^3+^ doping stabilizes the crystal lattice and suppresses Li^+^/Ni^2+^ cation mixing, thereby alleviating the intrinsic strain during lithiation and delithiation. Concurrently, the robust CeO_2_ coating acts as a protective layer, constraining particle expansion and dissipating stress. Together, these effects effectively mitigate internal stress and volume change, accounting for the superior structural stability observed in the Ce‐modified cathode material.

CV tests were carried out to study the redox behavior and reaction reversibility of the samples. Figure 6a illustrates that both the pristine and Ce‐modified NCM electrodes show typical redox pairs corresponding to the extraction and insertion of Li^+^ ions in the layered NCM structure. Notably, the potential difference (ΔV) between oxidation and reduction peaks decreases significantly from 0.18 V for the pristine NCM to 0.10 V for the NCM‐1% Ce, demonstrating enhanced electrochemical reversibility and reduced electrochemical polarization.

**FIGURE 6:**
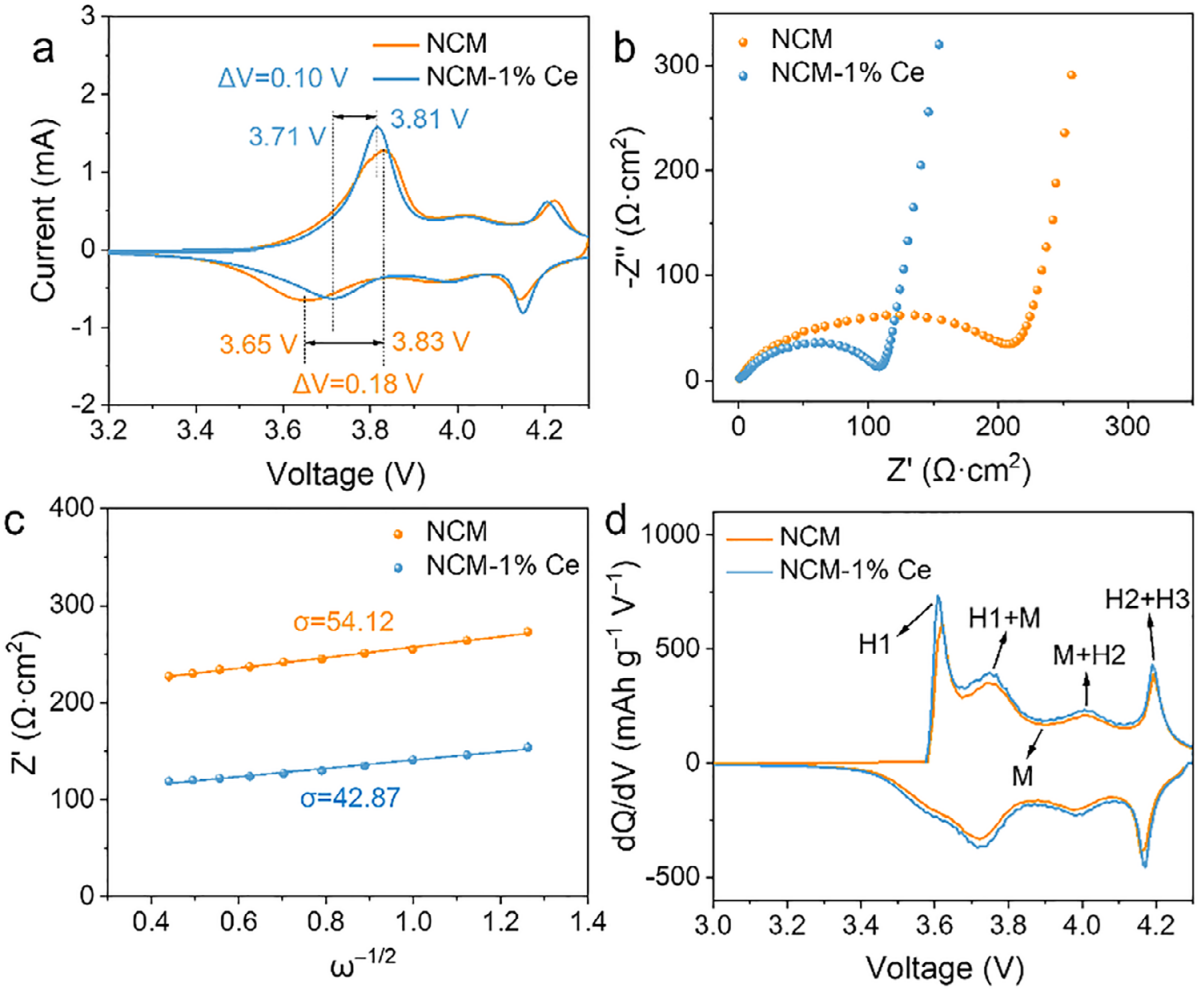
(a) CV curves, (b) Nyquist plots, (c) corresponding Z′ versus ω^−1/2^ plots, and (d) dQ/dV plots of the pristine NCM and NCM‐1% Ce.

EIS was further performed to investigate the interfacial resistance and Li^+^ diffusion kinetics. As displayed in Figure 6b and Table S6, the charge transfer resistance (*R_ct_*) of the NCM‐1% Ce electrode was calculated to be 70.59 Ω, which is much lower than that of the pristine NCM electrode (150.32 Ω), indicating a reduced interfacial resistance and improved charge transfer efficiency. In addition, the Warburg impedance factor (*σ*) was obtained from the relationship between the real part (*Z′*) and angular frequency *ω* ^−1/2^ according to Equation (1). Based on this (Figure 6c), the Li^+^ diffusion coefficient (D_Li+_) can be calculated by Equation (2).

(1) $$Z=\sigma \omega^{-1/2}\;$$

(2) $$D_{Li^{+}}=\frac{R^{2}T^{2}}{2A^{2}n^{4}F^{4}C^{2}\sigma^{2}}$$

where *R* stands for the gas constant, *T* stands for the absolute temperature, *A* stands for the area of the electrode material, n stands for the electron number exchanged during electrochemical reaction, *F* stands for the Faraday constant, and *C* is the molar concentration of Li^+^ ions. Fitting results indicate that the D_Li+_ value increases from 1.12 × 10^−14^ m^2^ s^−1^ for the pristine NCM material to 1.57 × 10^−14^ m^2^ s^−1^ for the NCM‐1% Ce material. The significantly reduced charge transfer resistance and enhanced Li^+^ diffusion efficiency contribute to the excellent cyclic stability and rate capability of the Ce‐modified NCM cathode.

For a more detailed analysis of the phase evolution of the materials upon cycling, the cycling curve was differentiated to generate the dQ/dV profile. The NCM cathode typically undergoes a series of complex phase transformations during the cycling process. As illustrated in Figure 6d, these transformations proceed sequentially from the hexagonal phase (H1) to the monoclinic phase (M), then to the second hexagonal phase (H2), and finally to the third hexagonal phase (H3). The peak intensity of each transition reflects the capacity contribution during discharge. Notably, the Ce‐modified cathode exhibits higher peak intensities across all phase transition stages, indicating that the modification enables a greater discharge capacity.

### 2.3 Gas Evolution Behavior

Considering the complexity of gas generation processes and possible overlap among gaseous products, the gas evolution behavior of pristine NCM and NCM‐1% Ce cathodes was investigated by DEMS (Figure S14). The cumulative amounts of the three major gaseous products (O_2_, H_2_, and CO_2_) detected after cycling were summed for comparative analysis, and the corresponding results are presented in Figure 7a,b. The pristine NCM battery exhibited obvious gas evolution behavior at all tested rates. During the initial activation process at 0.1 C, the cumulative gas release reached 3.30 µmol, with O_2_ accounting for the largest fraction among the detected gaseous species. This gas generation is likely associated with initial cathode–electrolyte interfacial reactions, electrolyte oxidation, and reactions involving surface residual species [53]. During subsequent cycling at 1, 2, and 5 C, the total gas evolution of pristine NCM reached 1.00, 0.79, and 0.63 µmol, respectively, with O_2_ remaining the primary gaseous product. The continuous gas evolution of pristine NCM indicates the occurrence of persistent interfacial degradation and electrolyte decomposition during cycling.

**FIGURE 7:**
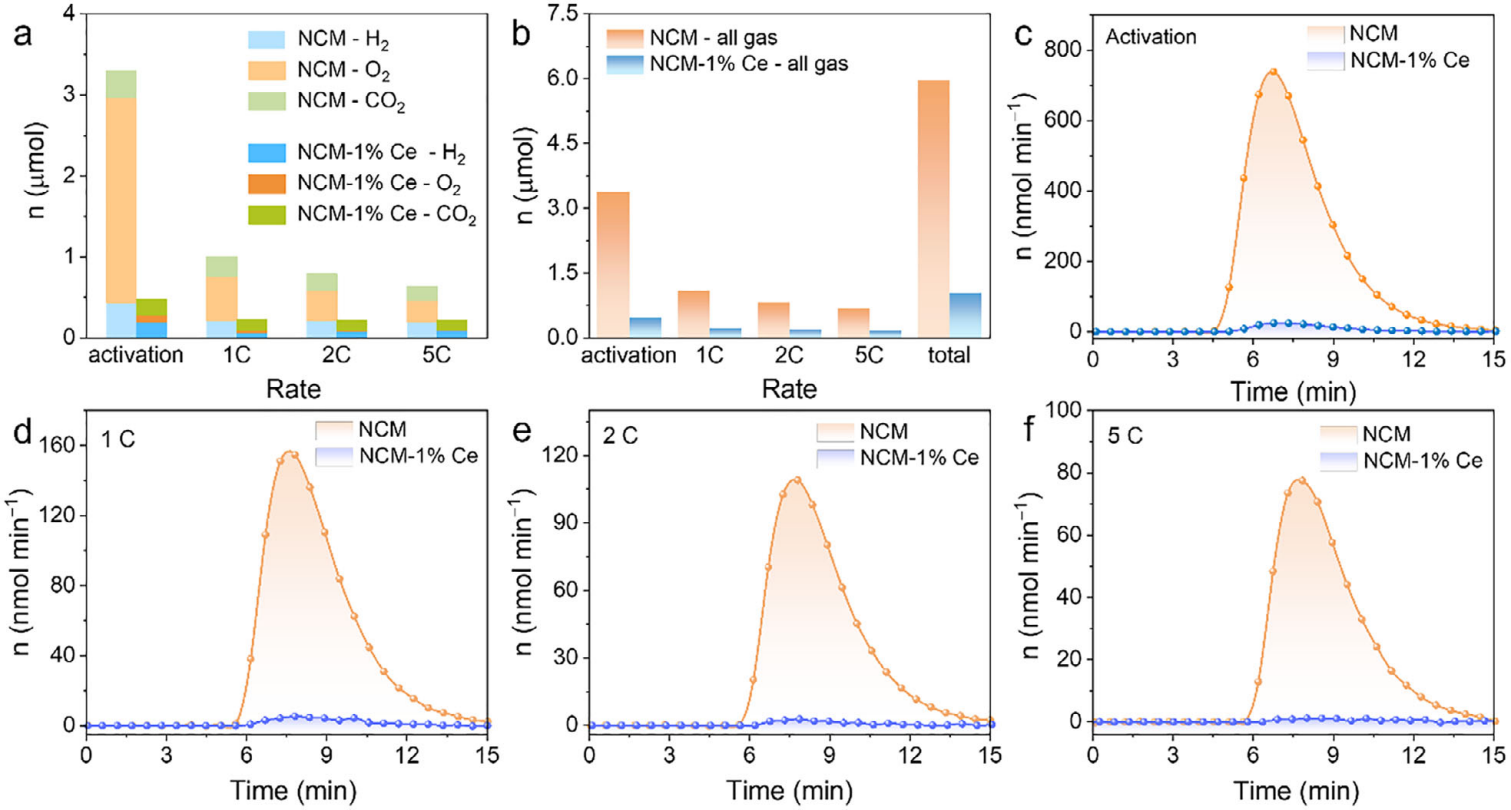
(a, b) Total gas evolution of the pristine NCM and NCM‐1% Ce at different rates. (c–f) O_2_ release profiles at different current densities.

In contrast, the NCM‐1% Ce system exhibited substantially suppressed gas evolution across the entire rate range. During activation, the total gas release of the NCM‐1% Ce sample was only 0.48 µmol, representing a reduction of approximately 86% compared with the pristine system, accompanied by a significant decline in the yield of each gas component. The total gas production at 1 C, 2 C, and 5 C was 0.23, 0.22, and 0.21 µmol, respectively, remaining nearly unchanged with increasing current density. Overall, the pristine NCM cathode released 5.72 µmol of gas under all tested conditions, approximately five times higher than that of NCM‐1% Ce (1.13 µmol). These results demonstrate that Ce modification effectively suppresses gas generation during both activation and cycling processes. The reduced gas evolution is attributed to the synergistic stabilization of the NCM lattice by Ce incorporation and suppression of cathode‐electrolyte interfacial reactions by the CeO_2_ surface coating.

Since O_2_ release from Ni‐rich cathodes is closely related to structural degradation and parasitic reactions, the O_2_ evolution behaviors of pristine NCM and NCM‐1% Ce were further analyzed, as shown in Figure 7c–f. Significant differences in O_2_ release capacity were observed between the two samples under all tested rates. During the activation of 0.1 C, the pristine NCM released 2539 nmol O_2_, while the NCM‐1% Ce released only 88 nmol, corresponding to a reduction of more than 96.5%. Similarly, remarkable suppression of O_2_ release was also achieved by the NCM‐1% Ce under high‐rate conditions. Under higher‐rate conditions, the O_2_ release amounts of the pristine NCM material reached 547, 385, and 272 nmol, respectively, whereas those of the NCM‐1% Ce material are drastically reduced to 20, 9, and 5 nmol, respectively. The significantly reduced O_2_ evolution demonstrates that Ce modification effectively enhances oxygen stability within the NCM cathode structure. The suppressed O_2_ release can further alleviate O_2_‐triggered electrolyte oxidation and interfacial degradation, contributing to improved chemical stability during cycling. To further evaluate the influence of Ce modification on electrolyte‐related gas generation, the cumulative release amounts of H_2_ and CO_2_ were analyzed, as shown in Figures S15 and S16. The NCM‐1% Ce cathode exhibited significantly lower H_2_ and CO_2_ evolution than pristine NCM during both activation and subsequent rate‐dependent cycling, confirming the effectiveness of the CeO_2_ coating in mitigating parasitic reactions at the cathode‐electrolyte interface.

### 2.4 Thermal Safety Performance Analysis

To verify the improvement in thermal safety of cathode materials before and after modification, accelerating rate calorimeter (ARC) was used to evaluate the adiabatic thermal runaway behaviors. 1 Ah NCM||Gr pouch cells with cathode dimensions of 56 mm × 43 mm were employed for the experiments. Figures S17 and S18 show the pouch cells before and after the ARC tests. And the corresponding results are presented in Figure 8. The pristine NCM shows an onset exothermic temperature (*T_1_*) of 130.62°C, a fast exothermic temperature (*T_2_*) of 170.19°C, and a peak thermal runaway temperature (*T_3_*) of 504.20°C. After modification, *T*_*1*_ increases to 135.32°C, *T*_*2*_ rises to 172.30°C, while *T*_*3*_ decreases to 495.00°C. Compared with the pristine NCM, the NCM‐1% Ce cathode material exhibits higher *T_1_* and *T_2_*, indicating that the modification layer effectively restrains side reactions between electrode and electrolyte and delays the thermal breakdown of the crystal structure. Furthermore, the longer duration between *T_1_* and *T_2_* for the NCM‐1% Ce material indicates a substantially reduced self‐heating rate, thereby providing sufficient response time and safety margin for thermal management. Meanwhile, the decreased *T_3_* weakens the intense heat release in the late stage of thermal decomposition. In general, the Ce modification strategy improves the thermal stability and adiabatic safety of the NCM cathode material, thus potentially reducing the risk of thermal runaway in LIBs under high‐temperature abuse conditions.

**FIGURE 8:**
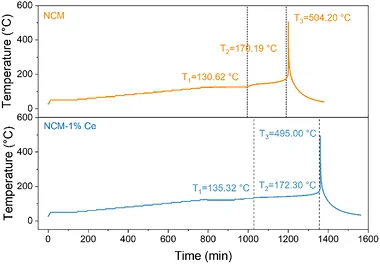
ARC thermal behavior curves of 1 Ah pristine NCM||Gr and NCM‐1% Ce||Gr pouch cells.

These results suggest that the synergistic Ce modification can be regarded as a versatile and robust strategy, which can be directly applied to various commercially synthesized NCM cathodes to simultaneously enhance their capacity, rate capability, and cycling stability, highlighting its great potential for practical industrial application.

## 3 Conclusions

Herein, Ce dual‐modified NCM811 cathode materials are prepared via a simple post‐synthetic modification strategy. The synergy between Ce^3+^ lattice doping and CeO_2_ surface coating efficiently reduces structural defects, inhibits cation mixing, and promotes Li^+^ transport kinetics, while also markedly suppressing side reactions at the cathode and electrolyte interface. As a result, the modified cathode achieves enhanced discharge capacity, outstanding cycling durability and excellent rate performance. After 500 cycles, the NCM‐1% Ce material exhibits a discharge capacity of 154.55 mAh g^−1^ and a capacity retention as high as 82.04%. Meanwhile, the modification greatly suppresses O_2_ release and gas evolution, and improves the adiabatic thermal safety verified by ARC measurement. This work reveals the synergistic optimization mechanism of Ce modification, and provides a promising route to develop high‐performance and high‐safety nickel‐rich cathode materials for advanced LIBs.

## 4 Experimental Section

### 4.1 Synthesis of the Modified NCM811 Materials

The Ce‐modified LiNi_0.8_Co_0.1_Mn_0.1_O_2_ (NCM‐Ce) material was prepared via a facile post‐synthetic solid‐state modification strategy. Commercial NCM powder (Shenzhen Kejing Co., Ltd.) and NCM‐R powder (Ronbay Technology Co., Ltd.) was thoroughly mixed with CeO_2_ nanoparticles (∼50 nm in diameter) in ethanol to form a homogeneous slurry under ultrasonic dispersion for 120 min. Four different weight fractions of CeO_2_ nanoparticles (0.5 wt.%, 1 wt.%, 1.5 wt.%, and 2 wt.% with respect to NCM) were mixed for modification. The resulting mixture was dried at 80°C overnight to remove the solvent, thereby yielding the precursor. The as‐prepared precursor was subsequently calcined in a tube furnace under a flowing N_2_/O_2_ mixed atmosphere (79:21, v/v). The temperature was raised to 550°C at a heating rate of 5°C min^−1^ and maintained for 12 h to promote the diffusion of Ce ions and the formation of a uniform CeO_2_ coating layer. After calcination, the product was washed with deionized water and ethanol at least three times to thoroughly remove residual impurities, followed by drying in a vacuum oven at 80°C for 12 h and sieving through a 200‐mesh sieve to obtain the final Ce‐modified NCM powder. The modified samples were named as NCM‐0.5% Ce, NCM‐1% Ce, NCM‐1.5% Ce, and NCM‐2% Ce, respectively.

### 4.2 Characterization Techniques

The crystal structures of the samples were analyzed by X‐ray diffraction (XRD, Thermo Scientific ARL EQUINOX 3500) with a Cu‐Kα radiation source. The diffraction patterns were collected over a 2θ range of 10°–80° with a step size of 0.01° and a scan speed of 5° min^−1^. X‐ray absorption fine structure (XAFS) spectroscopy was carried out using the RapidXAFS 2 M (Anhui Absorption Spectroscopy Analysis Instrument Co., Ltd.) in transmission mode at 20 kV and 20 mA, and the Si (553) spherically bent crystal analyzer with a radius of curvature of 500 mm was used for the Cu K‐edge. During the XAFS measurements, the position of the absorption edge was calibrated using a standard Cu foil sample, and all data were collected within a single time period. The surface morphologies of the cathode particles were observed using a scanning electron microscopy (SEM, Zeiss GeminiSEM 500). The microstructures and elemental distributions were obtained using transmission electron microscopy (TEM, Talos F200S) equipped with energy‐dispersive X‐ray spectroscopy (EDS, Oxford ULTIM Max 65). The surface chemical states were analyzed by X‐ray photoelectron spectroscopy (XPS, Thermo Scientific ESCALAB Xi+). The precise elemental concentrations were determined by inductively coupled plasma‐optical emission spectrometry (ICP‐OES, SPECTRO SPECTROBLUE FMX36).

### 4.3 Cell Assembly and Electrochemical Test

The cathodes were prepared by uniformly mixing the active material, super P conductive carbon black, and polyvinylidene fluoride (PVDF) binder in a weight ratio of 8:1:1 in *N‐*methyl‐*2*‐pyrrolidone (NMP) solvent to form a homogeneous slurry. The resulting slurry was cast onto aluminum foil current collectors using a doctor blade with an active material mass loading of 3 mg cm^−2^. The electrode was dried under vacuum at 105°C for 12 h to remove the residual solvent. CR2016 coin cells were assembled to evaluate the electrochemical performance of the pristine and Ce‐modified NCM cathodes. Half cells using lithium metal as the anode were assembled in a glove box filled with argon gas, with the content of both water and oxygen being less than 1 ppm. The electrolyte was composed of 1.2 M LiPF_6_ dissolved in a mixture of ethylene carbonate (EC), diethyl carbonate (DEC), and dimethyl carbonate (DMC) with a volume ratio of 1:1:1, supplemented with 1 wt.% vinylene carbonate (VC) and 1 wt.% fluoroethylene carbonate (FEC) as functional additives.

The galvanostatic charge/discharge measurements were carried out on a LAND CT3002A battery testing system within a voltage window of 2.75–4.2 V (vs. Li/Li^+^) at different current densities, where 1 C was defined according to the capacity of 200 mAh g^−1^. All tests of electrochemical performance were performed in a thermostatic chamber at 25°C. Cyclic voltammetry (CV) tests were performed on a CHI 760E electrochemical workstation (CH Instrument Inc.) over a voltage range of 3–4.3 V at a scan rate of 0.1 mV s^−1^. Electrochemical impedance spectroscopy (EIS) was performed on the same electrochemical workstation, over a frequency range from 0.1 Hz to 100 kHz with an AC amplitude of 5 mV, and the corresponding EIS data were fitted and analyzed using ZView II software.

### 4.4 Gas Evolution Behavior Test

Gas evolution analysis was performed using differential electrochemical mass spectrometry (DEMS, Hiden HPR‐20).The DEMS system consists of a carrier gas supply unit, an electrochemical cell, and a quadrupole mass spectrometer. High‐purity argon (Ar, 99.999%) was employed as the carrier gas to transport gaseous byproducts generated during electrochemical reactions into the mass spectrometer. A cold trap maintained at −30°C was used to remove volatile electrolyte components and reduce background interference. By combining electrochemical signals with mass spectrometric detection, DEMS enables qualitative identification and quantitative analysis of gaseous products. The DEMS setup has been described in detail elsewhere [54, 55].

The custom‐designed DEMS cell was assembled in an Ar‐filled glove box using lithium metal as the anode and glass fiber as the separator. The sealed cell was connected to a Land battery testing system, and galvanostatic cycling was conducted at 25°C with an upper cutoff voltage of 4.5 V. The testing protocol consisted of one formation cycle at 0.1 C, followed by one single cycle at each of 1, 2, and 5 C (1 C = 220 mA g^−1^). After each individual cycle at a given rate, the evolved gaseous products were analyzed by DEMS, and the cumulative gas generation amount for that specific cycle was quantified. Before measurement, the gas pipeline was purged with high‐purity Ar at a flow rate of 1 mL min^−1^ for 15 min to stabilize the baseline. Gas calibration was performed using a standard gas mixture containing 100 ppm H_2_, 100 ppm O_2_, and 100 ppm CO_2_ with ultrahigh‐purity Ar (99.9999%) as the balance gas. The gas species were monitored based on their characteristic mass‐to‐charge ratios (m/z) 2, 32, 44, and 40 corresponding to H_2_, O_2_, CO_2_, and Ar, respectively. The total gas production was finally obtained by integrating the gas evolution profiles.

### 4.5 Thermal Runaway Test

Thermal runaway tests were performed on 1 Ah pouch cells using an accelerating rate calorimeter (ARC, BTC‐130, HEL Ltd., UK). Cathodes were fabricated following the aforementioned procedure and cut into rectangular pieces with dimensions of 56 mm × 43 mm. For pouch cell assembly, graphite anodes were matched with the corresponding cathodes at a negative‐to‐positive (N/P) capacity ratio of 1.1. The cells were first fully charged with constant‐current charging at 1 C until the cell voltage rose to 4.2 V, followed by constant‐voltage charging at 4.2 V until the current dropped to 0.02 C. The charged cells were then positioned at the center of the ARC chamber. The Heat‐Wait‐Search (HWS) mode was activated throughout the test to monitor self‐heating reactions upon each temperature ramp. The initial temperature was set to 50°C, with a 5°C temperature increment for each heating segment followed by an equilibration waiting period. The self‐heating detection threshold of the ARC was set to 0.02°C min^−1^. When the self‐heating rate exceeded this threshold, the calorimeter automatically switched to the exothermic tracking mode.

## Author Contributions

**Wei‐Wei Wang**: methodology, data curation, investigation, validation, formal analysis, writing – original draft, writing – review and editing, project administration. **Da‐Kun Chen**: data curation, investigation, validation, formal analysis, writing – original draft. **Wei Kang**: methodology, data curation, investigation, validation, formal analysis, writing – original draft. **Xiao‐Mei Wu**: data curation, investigation, validation, formal analysis, writing – original draft. **Qing Cao**: data curation, investigation, validation, formal analysis, writing – original draft. **Quan‐Fang Wang**: methodology, data curation, investigation, validation, formal analysis, writing – original draft. **Si‐Yuan Ma**: data curation, investigation, validation, formal analysis, supervision, funding acquisition, writing – review and editing, writing – original draft, resources. **Hai‐Long Wang**: data curation, investigation, validation, formal analysis, supervision, writing – review and editing, resources. **Jing‐Hua Tian**: conceptualization, methodology, data curation, investigation, validation, formal analysis, supervision, funding acquisition, project administration, writing – original draft, writing – review and editing, resources. **Zhong‐Qun Tian**: funding acquisition, supervision, writing – review and editing, resources.

## Funding

This work was supported by the Fujian Provincial Natural Science Foundation (2025J011016, 2024J011449), the National Natural Science Foundation of China (22372072), the Natural Science Foundation of Xiamen (3502Z202471082), and the Fundamental Research Funds for the Central Universities (20720250005). The AI‐driven experiments, simulations, and model training were performed on the robotic AI‐Scientist platform of the Chinese Academy of Sciences.

## Conflicts of Interest

The authors declare no conflicts of interest.

## Supporting information




**Supporting File**: advs78626‐sup‐0001‐SuppMat.docx.

## Data Availability

The data that support the findings of this study are available from the corresponding author upon reasonable request.
