# Supplementary material for: Post‐Synthetic Cerium Modification of Commercial NCM811 for Concurrent Cycling and Safety Upgrading
Source: Adv Sci (Weinh). 2026 Nov 3:e78626. Online ahead of print. doi: 10.1002/advs.78626 (PMC13731070; doi:10.1002/advs.78626)
Supplement: Supplementary file 1 [file ADVS-9999-e78626-s001.docx]

Supporting Information

**Post-Synthetic Cerium Modification of Commercial NCM811 for Concurrent Cycling and Safety Upgrading**

Wei-Wei Wang^1^, Da-Kun Chen^1,2^, Wei Kang^1^, Xiao-Mei Wu^2^, Qing Cao^2^, Quan-Fang Wang^2^, Si-Yuan Ma^1,2,^*, Hai-Long Wang^1,2,^*, Jing-Hua Tian^1,2,^*, Zhong-Qun Tian^1,2^

**Figure S1**. XRD patterns of the NCM samples with different Ce contents; (b) enlarged view of the 28.8° diffraction peak in (a).

**Figure S2**. SEM images of (a) the pristine NCM, (b) NCM-0.5 wt% Ce, (c) NCM-1 wt% Ce, (d) NCM-1.5 wt% Ce, and (e) NCM-2 wt% Ce samples.

**Figure S3**. (a) Nyquist plots and (b) the comparison of fitted impedance parameters (R_S_, R_SEI_, and R_ct_) obtained from equivalent circuit fitting for the modified NCM with different Ce contents.

**Figure S4.** Equivalent circuit model used for fitting the EIS data.

**Figure S5.** (a) The enlarged (003) peak of NCM and NCM-1% Ce samples. High-resolution XPS spectra of (b) Co 2p and (c) Mn 2p of NCM and NCM-1% Ce samples.

**Figure S6**. Crystal structure of the pristine NCM and NCM-1% Ce.

**Figure S7**. (a)Ni, (b) Co, and (c) Mn *K*-edge XANES spectra with first derivative of the pristine NCM and NCM-1% Ce. (d)Ni, (e) Co, and (f) Mn *K*-edge EXAFS spectra of the pristine NCM and NCM-1% Ce.

**Figure S8**. (a) XPS survey spectra and (b) Ce 4d high-resolution XPS spectra of the NCM-1% Ce obtained by sputtering at different depths.

**Figure S9.** HRTEM image of the (a) pristine NCM and (b) NCM-1% Ce.

**Figure S10**. Initial charge and discharge profiles of LFP||Li cells at 1C under (a) a high cut-off voltage of 4.5 V and (b) a high temperature of 60 °C. Long-term cycling performance of the LFP||Li cells at 1C under (c) a high cut-off voltage of 4.5 V and (d) a high temperature of 60 °C.

**Figure S11.** (a) Initial charge and discharge profiles at 0.1C, (b) rate capability and (c) long-term cycling performance at 1C of the pristine NCM-R and NCM-R-1% Ce.

**Figure S12.** Schematic diagram of lattice regulation on c-axis parameter, transition metal slab spacing and Li interlayer spacing of NCM cathode via strong Ce-O bonding after Ce doping.

**Figure S13.** SEM images of the(a) pristine NCM and (b) NCM-1% Ce after 500 cycles at 1C.

**Figure S14**. Schematic diagram of DEMS system.

**Figure S15.** H_2_ release profiles at different rates. (a) after activation, (b) 1C, (c) 2C, (d) 5C, and (e) Total.

**Figure S16.** CO_2_ release profiles at different rates. (a) after activation, (b) 1C, (c) 2C, (d) 5C, and (e) Total.

**Figure S17.** Comparison of the NCM battery states before and after ARC testing: (a) initial state before testing; (b) state after testing.

**Figure S18.** Comparison of the NCM-1% Ce battery states before and after ARC testing: (a) initial state before testing; (b) state after testing.

**Table S1.** The ICP-OES results of the pristine NCM and NCM-1% Ce samples.

| Samples | Li (wt%) | Ni (wt%) | Co (wt%) | Mn (wt%) | Ce (wt%) |
| --- | --- | --- | --- | --- | --- |
| Pristine NCM | 7.15 | 52.31 | 3.02 | 7.72 | / |
| NCM-1% Ce | 7.04 | 51.27 | 2.96 | 7.49 | 0.79 |

**Table S2.** XRD Rietveld refinement parameters of the pristine NCM.

| Name | Type | x | y | z | frac |
| --- | --- | --- | --- | --- | --- |
| Li1 | Li^+^ | 0 | 0 | 0.5 | 0.9686 |
| Ni1 | Ni^2+^ | 0 | 0 | 0.5 | 0.0314 |
| Li2 | Li^+^ | 0 | 0 | 0 | 0.0314 |
| Ni2 | Ni^2+^ | 0 | 0 | 0 | 0.7911 |
| Co | Co^3+^ | 0 | 0 | 0 | 0.1289 |
| Mn | Mn^4+^ | 0 | 0 | 0 | 0.0487 |
| O | O^2–^ | 0 | 0 | 0.2558 | 1 |

**Table S3.** XRD Rietveld refinement parameters of the NCM-1% Ce.

| Name | Type | x | y | z | frac |
| --- | --- | --- | --- | --- | --- |
| Li1 | Li^+^ | 0 | 0 | 0.5 | 0.9893 |
| Ni1 | Ni^2+^ | 0 | 0 | 0.5 | 0.0107 |
| Li2 | Li^+^ | 0 | 0 | 0 | 0.0107 |
| Ni2 | Ni^2+^ | 0 | 0 | 0 | 0.8010 |
| Ce1 | Ce^3+^ | 0 | 0 | 0 | 0.0067 |
| Co | Co^3+^ | 0 | 0 | 0 | 0.1285 |
| Mn | Mn^4+^ | 0 | 0 | 0 | 0.0538 |
| O | O^2–^ | 0 | 0 | 0.2592 | 1 |

**Table S4.** Rietveld-refined lattice parameters of the pristine NCM and NCM-1% Ce samples.

| **Lattice constant** | **a (Å)** | **b (Å)** | **c (Å)** | **V (Å^3^)** | **Li^+^/Ni^2+^ cation mixing** |
| --- | --- | --- | --- | --- | --- |
| NCM | 2.881 | 2.881 | 14.233 | 102.327 | 3.13% |
| NCM-1% Ce | 2.864 | 2.856 | 14.149 | 100.517 | 1.07% |

**Table S5.** Comparison of the radii of different metal ions.

| **Metal ions** | **Li^+^** | **Ni^2+^** | **Co^3+^** | **Mn^4+^** | **Ce^3+^** |
| --- | --- | --- | --- | --- | --- |
| Ionic radii (**Å**) | 0.76 | 0.69 | 0.55 | 0.53 | 1.02 |

**Table S6.** EIS parameters obtained from equivalent circuit fitting for the pristine NCM and NCM-1% Ce cells.

| **Samples** | **R_S_ (Ω)** | **R_SEI_ (Ω)** | **R_ct_ (Ω)** | **D_Li+_ (m^2^ s^–1^)** |
| --- | --- | --- | --- | --- |
| NCM | 2.04 | 42.16 | 150.32 | 1.12 × 10^–14^ |
| NCM-1% Ce | 2.09 | 40.09 | 70.59 | 1.57 × 10^–14^ |
